# Acute Minocycline Treatment Mitigates the Symptoms of Mild Blast-Induced Traumatic Brain Injury

**DOI:** 10.3389/fneur.2012.00111

**Published:** 2012-07-16

**Authors:** Erzsebet Kovesdi, Alaa Kamnaksh, Daniel Wingo, Farid Ahmed, Neil E. Grunberg, Joseph B. Long, Christine E. Kasper, Denes V. Agoston

**Affiliations:** ^1^U.S. Department of Veterans Affairs, Veterans Affairs Central OfficeWashington, DC, USA; ^2^Department of Anatomy, Physiology and Genetics, School of Medicine, Uniformed Services UniversityBethesda, MD, USA; ^3^Center for Neuroscience and Regenerative Medicine at the Uniformed Services UniversityBethesda, MD, USA; ^4^Department of Medical and Clinical Psychology, School of Medicine, Uniformed Services UniversityBethesda, MD, USA; ^5^Blast-Induced Neurotrauma Branch, Center for Military Psychiatry and Neuroscience, Walter Reed Army Institute of ResearchSilver Spring, MD, USA

**Keywords:** TBI, anti-inflammatory, treatment, neurobehavior, proteomics

## Abstract

Mild traumatic brain injury (mTBI) represents a significant challenge for the civilian and military health care systems due to its high prevalence and overall complexity. Our earlier works showed evidence of neuroinflammation, a late onset of neurobehavioral changes, and lasting memory impairment in a rat model of mild blast-induced TBI (mbTBI). The aim of our present study was to determine whether acute treatment with the non-steroidal anti-inflammatory drug minocycline (Minocin^®^) can mitigate the neurobehavioral abnormalities associated with mbTBI, Furthermore, we aimed to assess the effects of the treatment on select inflammatory, vascular, neuronal, and glial markers in sera and in brain regions associated with anxiety and memory (amygdala, prefrontal cortex, ventral, and dorsal hippocampus) following the termination (51 days post-injury) of the experiment. Four hours after a single exposure to mild blast overpressure or sham conditions, we treated animals with a daily dose of minocycline (50 mg/kg) or physiological saline (vehicle) for four consecutive days. At 8 and 45 days post-injury, we tested animals for locomotion, anxiety, and spatial memory. Injured animals exhibited significantly impaired memory and increased anxiety especially at the later testing time point. Conversely, injured and minocycline treated rats’ performance was practically identical to control (sham) animals in the open field, elevated plus maze, and Barnes maze. Protein analyses of sera and brain regions showed significantly elevated levels of all of the measured biomarkers (except VEGF) in injured and untreated rats. Importantly, minocycline treatment normalized serum and tissue levels of the majority of the selected inflammatory, vascular, neuronal, and glial markers. In summary, acute minocycline treatment appears to prevent the development of neurobehavioral abnormalities likely through mitigating the molecular pathologies of the injury in an experimental model of mbTBI.

## Introduction

Traumatic brain injury (TBI) is a prominent health concern worldwide as it is one of the major causes of death and chronic disability (Hyder et al., 2007). The mild form of traumatic brain injury (mTBI) has become an especially significant challenge for the civilian (Thurman et al., 1999) and the military healthcare systems (Hoge et al., 2008; Tanielian and Jaycox, 2008) due to its high prevalence and the absence of serious acute symptoms following injury. Blast-induced mTBI (mbTBI) was the most frequent form of mTBIs sustained during recent military conflicts (Warden, 2006; Terrio et al., 2009). There is currently no objective diagnosis for mbTBI, a minimal understanding of its underlying pathologies, and consequently a lack of specific, evidence based treatments.

Symptoms of blast-induced TBI (bTBI) include increased anxiety as well as memory impairment that may not be detectable for weeks or months after the exposure (Ryan and Warden, 2003; Okie, 2005; Nelson et al., 2009; Terrio et al., 2009; Cernak and Noble-Haeusslein, 2010; Hoffer et al., 2010). The delayed onset of neurobehavioral impairments suggests a lasting secondary injury process involving distinct brain regions (Moser and Moser, 1998). The ventral hippocampus (VHC) along with the prefrontal cortex (PFC) and the amygdala (AD) are involved in mediating anxiety, while the dorsal hippocampus (DHC) is involved in mediating spatial learning and memory (Henke, 1990; Moser and Moser, 1998; Bremner, 2005, 2007). Using a rat model of bTBI, we found that a single mild blast overpressure exposure results in increased anxiety and memory impairment (Kovesdi et al., 2011; Kwon et al., 2011). Importantly, the memory impairment was not detectable within the first week of the exposure; it became significant 2 weeks post-injury and persisted for at least 2 months after (Kovesdi et al., 2011; Kwon et al., 2011).

Our immunohistochemical and proteomics analyses of these animals showed evidence of neuronal and glial cell loss, gliosis, and neuroinflammation at 2 months post-injury. In addition to an increased presence of microglia in the DHC and the VHC of injured animals as well as increased tissue levels of interleukin-6 (IL-6) and interferon-gamma (IFNγ) in these brain regions. Neuroinflammation can adversely affect neuronal function by directly causing neuronal cell death as well as increasing neuron vulnerability to noxious factors like excitotoxins, which are also elevated after injury (Arvin et al., 1996; Morganti-Kossmann et al., 2002; Cacci et al., 2005; Floyd and Lyeth, 2007; Kochanek et al., 2008; Agoston et al., 2009; Agostinho et al., 2010; Czlonkowska and Kurkowska-Jastrzebska, 2011; Robel et al., 2011). Based on our previous evidence linking neuroinflammation to neurobehavioral abnormalities (Kovesdi et al., 2011), we hypothesized that anti-inflammatory treatment may improve the functional outcome in mbTBI.

To test our hypothesis, we selected the anti-inflammatory drug minocycline for several reasons. Minocycline hydrochloride easily crosses the blood brain barrier (BBB), is well characterized, safe, FDA approved, and has been used experimentally and clinically (Macdonald et al., 1973; Saivin and Houin, 1988). Similar to its tetracycline analogs, the side effects of minocycline treatment are mild and include discoloration of the teeth, gastrointestinal irritability, and candidiasis (Fanning et al., 1977; Gump et al., 1977). In humans, long-term treatment is generally safe and is well tolerated up to 200 mg/day. In animals, the lethal dose of minocycline is very high at 3600 mg/kg (Blum et al., 2004); the “therapeutic” dosage utilized in animal experiments ranges between 10 and 90 mg/kg with an average of 50 mg/kg for daily treatments (e.g., Wells et al., 2003; Stirling et al., 2004; Festoff et al., 2006; Li and McCullough, 2009; Abdel Baki et al., 2010; Lee et al., 2010; Siopi et al., 2011; Wixey et al., 2011; Ng et al., 2012).

Minocycline has been successfully used in various animal models of brain and spinal cord injuries as well as neurodegenerative diseases like Huntington’s (Blum et al., 2004), where it was shown to reduce tissue damage and inflammation, and improve neurological outcome (Yrjanheikki et al., 1999; Chen et al., 2000; Kriz et al., 2002; Wu et al., 2002; Wells et al., 2003; Xu et al., 2004; Zemke and Majid, 2004; Festoff et al., 2006; Marchand et al., 2009). Using a rat model of mbTBI, we report that acute treatment with minocycline mitigates the inflammatory response to injury and results in normalized neurobehavior.

## Materials and Methods

### Experimental groups and housing conditions

Thirty-two male Sprague Dawley rats (Charles River Laboratories, Wilmington, MA, USA) were used, weighing 245–265 g at the beginning of the experiment. All animals were kept under normal housing conditions (two rats/cage) in a reverse 12–12 h light-dark cycle and provided with food and water *ad libitum* for the entire length of the study. Following baseline behavioral testing (described below), animals were assigned to one of the following experimental groups: (1) sham saline treated (*sham-vehicle*; *n* = *8*) and (2) sham minocycline treated (*sham-mino*; *n* = 8), which served as controls for (3) blast injured saline treated (*injured-vehicle*; *n* = 8) and (4) blast injured-minocycline treated (*injured-mino*; *n* = 8), respectively. All animals were handled according to protocol approved by the Institutional Animal Care and Use Committee (IACUC) at the Uniformed Services University (USU).

### Behavioral tests

**Figure A1:**
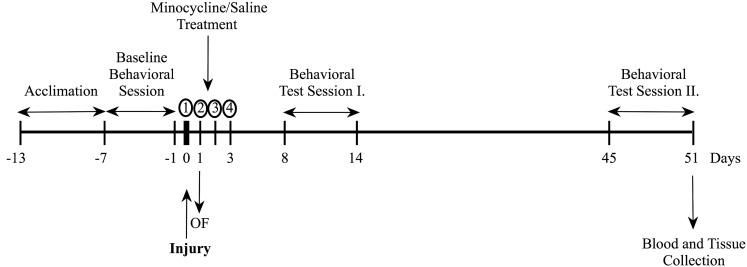
**Outline of the experimental schedule**. After 1 week of acclimation, baseline behavioral analyses, and injury (or sham), rats were treated intraperitoneally for four consecutive days (marked by circled numbers of 1 through 4) with 50 mg/kg of minocycline or saline starting at 4 hours after injury. Behavioral assessments (Open Field, Elevated Plus Maze, and Barnes Maze) were conducted before injury (Baseline Behavioral Session), and at 1 (open field OF only), 8 (Behavioral Test Session I.), and 45 days (Behavioral Test Session II.) after injury or sham. At the end of the experiment blood and brains were collected, processed, and analyzed using RPPM and ELISA.

Prior to injury, all rats underwent baseline behavioral assessments for general locomotor activity by the open field (OF) test, and for anxiety by the elevated plus maze (EPM). Rats were also trained for five consecutive days in the Barnes maze (BM) for spatial learning and memory. The baseline test results (data not shown) were used to create the aforementioned experimental groups with no statistical significance among them. Following injury or sham, two behavioral test sessions were conducted starting at 8 and 45 days. The experimental schedule is illustrated in Figure A1 in Appendix. Within each testing session, the behavioral tests were performed on separate days in the following order: OF (day 1), EPM (day 2), and BM (days 3–7). All behavioral tests were performed during animals’ dark cycle.

#### Open field

Tests were performed using AccuScan’s infrared light beams OF system (AccuScan Instruments, Inc.) at baseline and 1, 8, and 45 days post-injury. The OF system is a 16.5 × 16.5 × 13 (L × W × H) inches clear Plexiglas arena with a perforated lid. The system uses 16 × 16 grid light beam arrays in the *X* and *Y* axes to measure locomotor activity. The system detects beam breaks by the animal and determines the location of the rat within the cage. During the 60 min testing period, horizontal activity (number of beam breaks) and resting time (time spent with inactivity greater than or equal to 1 s) were measured. Data for each animal were recorded and analyzed automatically with Fusion 3.4 software (AccuScan Instruments, Inc.). The horizontal activity and resting time are presented as the average performance of all animals in each experimental group ±standard error of the mean (SEM) at each of the individual time points.

#### Elevated plus maze

The EPM is an ethologically relevant assessment of anxiety levels in rodents (Carobrez and Bertoglio, 2005; Salzberg et al., 2007; Walf and Frye, 2007). Tests were carried out prior to injury and at 9 and 46 days post-injury as described earlier in details (Kovesdi et al., 2011). Briefly, rats were placed one by one in the center of the maze facing one of the open arms. During the 5 min testing session, each animal was allowed to explore the maze freely while its movement was video-tracked. Time spent in the open and the closed arms (seconds) was recorded for each animal using ANY-maze 4.2 Software (Stoelting Company, Wood Dale, IL, USA). The maze was cleaned with a 30% ethanol solution between each trial. Data are presented as the average time (in seconds) spent in the open vs. the closed arms of the maze in each experimental group ±SEM.

#### Barnes maze

Barnes maze represents a widely used and less stressful alternative to the Morris water maze for assessing spatial memory in rodents (Barnes, 1979; Maegele et al., 2005; Doll et al., 2009; Harrison et al., 2009). Tests were carried out prior to injury (training session), and at 10 and 47 days post-injury (Test Session I and II, respectively; Kovesdi et al., 2011). The maze is a circular platform (1.2 m in diameter) that contains 18 evenly spaced holes around the periphery. One of the holes is the entrance to a darkened escape box that is not visible from the surface of the board. The position of the escape chamber relative to the other holes and the testing room remains fixed during all BM trials. On the first day of the training session, each rat was placed in the escape box and covered for 30 s. The escape box was then removed with the animal inside and moved to the center of the maze. The rat was allowed to explore the maze for a few seconds after which it was returned to its home cage. In the second and third trial (only day 1 of the BM training session has three trials), the same rat was placed under a start box in the center of the maze for 30 s. The start box was removed and the rat was allowed to explore freely to find the escape box. Training sessions ended after the animal had entered the escape box or when a pre-determined time (240 s) had elapsed. If the animal had not found the escape box during the given time period, it was placed in the escape box for 1 min at the end of the trial. During the baseline BM session, animals were trained until their daily latency time averaged 10 s. The two post-injury BM test sessions were run for five consecutive days; every rat was tested twice per day as described above. In each trial, the latency to enter the escape box was measured and recorded using ANY-maze 4.2 Software (Stoelting Company, Wood Dale, IL, USA). The escape box and the maze were cleaned with a 30% ethanol solution between each trial and animal. Data are presented as the average latency times of two daily trials per animal per experimental group ±SEM.

### Mild blast injury

On the day of the injury all rats (average weight ~300 g) were transferred to Walter Reed Army Institute of Research (Silver Spring, MD, USA) as described in detail (Kamnaksh et al., 2011). Sixteen rats were exposed to whole body mbTBI as described earlier (Long et al., 2009; Kovesdi et al., 2011; Kwon et al., 2011). Briefly, rats were anesthetized with 4% Isoflurane for 6 min in an induction chamber (Forane, Baxter Healthcare Corporation, Deerfield, IL, USA), placed in an animal holder within the shock tube in a transverse prone position, and exposed to whole body blast overpressure (20.6 ± 3 psi) while wearing chest protection. The other 16 rats were similarly anesthetized, placed in the shock tube, but were not exposed to blast overpressure (sham). Following blast injury or sham, rats were moved back to their home cages and transported back to the USU animal facility.

### Pharmacological treatment

Four hours after injury or sham, rats received a total volume of 0.25 ml/100 g body weight of either physiological saline alone (*vehicle*) or 50 mg/kg of clinical grade minocycline (Minocin^®^, Triax Pharmaceuticals, Italy) dissolved in saline (*mino*) intraperitoneally (i.p.). Animals received minocycline or saline for four consecutive days at identical times each day. Our minocycline dosage and treatment paradigm was based on previous studies using rodent models of various neurological conditions where minocycline was administered i.p. at an average dose of 50 mg/kg (see Table A1 in Appendix).

### Tissue collection and processing

At the completion of the last behavioral test session (51 days post-injury or sham), animals were placed inside an induction chamber saturated with Isoflurane and deeply anesthetized until a tail pinch produced no reflex movement. Anesthesia was maintained using a mask/nose cone attached to the anesthetic vaporizer and blood was collected (1.5 ml) from a tail vein; serum was prepared as described earlier (Kwon et al., 2011). For measuring tissue levels of protein markers, rats were decapitated and brains were immediately removed and placed on ice. The amygdala (AD), PFC, VHC, and DHC were dissected, frozen, and stored at −80°C until use as described earlier (Kwon et al., 2011).

#### Protein measurements

Sample preparation, printing, scanning, and data analysis of serum and brain regions were performed using Reverse Phase Protein Microarray (RPPM) as described earlier (Kovesdi et al., 2011; Kwon et al., 2011). Briefly, frozen brain tissues were pulverized in liquid nitrogen, the powder was transferred into a lysis buffer (Thermo Fisher, Waltham, MA, USA) with protease and phosphatase inhibitors (Thermo Fisher), sonicated, centrifuged, and the supernatants aliquoted and stored at −80°C. Protein concentrations were measured by BCA assay (Thermo Fisher). Blood samples were centrifuged at 10,000 × *g* for 15 min at 4°C; supernatants were aliquoted, flash-frozen, and stored at −80°C.

Tissue samples were diluted in print buffer and then subjected to an 11-point serial 1:2 dilution and transferred into Genetix 384-well plates (X7022, Fisher Scientific, Pittsburg, PA, USA) using a JANUS Varispan Integrator and Expanded Platform Workstation (PerkinElmer, Waltham, MA, USA). Plates were transferred into an Aushon 2470 Arrayer (Aushon Biosystem, Billerica, MA, USA) to be printed on ONCYTE Avid (brain samples) or ONCYTE Nova (serum samples) single-pad nitrocellulose coated glass slides (Grace Bio-Labs, Bend, OR, USA; Gyorgy et al., 2010).

Primary antibodies (Table A2 in Appendix) were diluted to 10× the optimal Western analysis concentration in antibody incubation buffer as described earlier (Gyorgy et al., 2010). The primary antibody solution was incubated overnight at 4°C with a cover slip. The following day slides were washed and then incubated with an Alexa Fluor^®^ 635 goat anti-mouse (Cat# A-31574), goat anti-rabbit (Cat# A-31576), or rabbit anti-goat IgG (H + L; Cat# A-21086) secondary antibodies from Invitrogen at 1:6000 dilution in antibody incubation buffer for 1 h at room temperature. After washing and drying, fluorescent signals were measured by a Scan Array Express HT microarray scanner (Perkin Elmer, Waltham, MA, USA) using a 633 nm wavelength laser and a 647 nm filter.

Data from the scanned images were imported into a Microsoft Excel-based bioinformatics program developed in-house for analysis (Gyorgy et al., 2010). The linear regression of the log–log data was calculated after the removal of flagged data, which include signal to noise ratios of less than 2, spot intensities in the saturation range or noise range, or high variability between duplicate spots (>10–15%). The total amount of antigen is determined by the *y*-axis intercept (*Y*-cept; Gyorgy et al., 2010). Data is reported as the mean *Y*-cept ±SEM.

#### Corticosterone assay

Serum corticosterone (CORT) levels were measured with Cayman’s Corticosterone EIA Kit according to the manufacturer’s instructions (Cayman Chemical, Ann Arbor, MI, USA). Each sample was diluted 1:500 and measured in triplicate (Kwon et al., 2011). Data is reported as the mean concentration (in pg/mg) ±SEM.

### Statistical analysis

All data were analyzed using Graph Pad Instat software (GraphPad Software, Inc., La Jolla, CA, USA). Statistical significance was verified by one-way analysis of variance (ANOVA), followed by Tukey *post hoc* test for multiple comparison. Differences with a *p* value of <0.05 were considered significant.

## Results

### Behavioral tests

**Figure 1:**
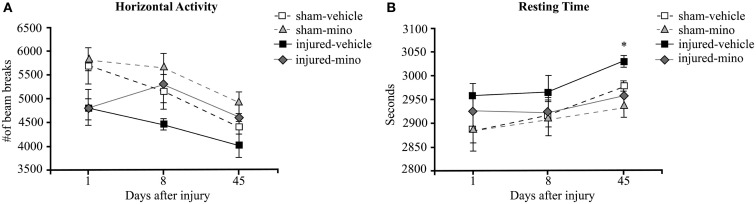
**The effect of injury and minocycline treatment on basic locomotor activities at different time points after mbTBI**. **(A)** Horizontal activity (number of beam breaks), and **(B)** Resting time (seconds) were measured in Open field. Data are presented as mean ± SEM. *p < 0.05 for injured-vehicle vs. sham-mino rats.

One day following blast exposure, injured rats showed reduced horizontal activity and slightly increased resting time in the OF compared to sham animals, but the differences were not statistically significant (Figure 1A). At 8 days post-injury, the horizontal activity of injured-vehicle animals further decreased. On the other hand, injured-mino rats had a similar horizontal activity to animals in the two sham groups. The horizontal activity of animals in all groups was the lowest at 45 days after injury. Similarly, animals in all experimental groups spent more time resting with injured-vehicle animals spending significantly more time resting than animals in the other three groups (Figure 1B).

**Figure 2:**
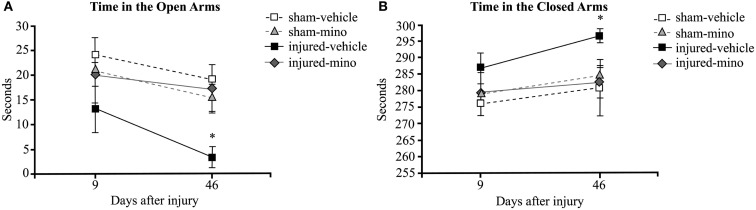
**The effect of injury and minocycline treatment on anxiety levels at different time points after mbTBI**. **(A)** Time spent in the open arms (seconds), and **(B)** time spent in the closed arms (seconds) were measured for all animals in the elevated plus maze. Data are presented as mean ± SEM. *p < 0.05 for injured-vehicle vs. sham-vehicle rats.

During the first EPM testing performed 9 days after exposure, injured-vehicle animals spent less time in the open arms and more time in the closed arms of the maze than animals in the other three groups (Figures 2A,B). However, the difference at this time point was not statistically significant. At 46 days after injury, the differences in the time spent in the open and closed arms of the maze became significant between injured-vehicle and injured-mino animals. At this later time point, injured-vehicle animals barely spent any time in the open arms of the maze and practically spent all of their time in the closed arms of the maze (Figures 2A,B). By contrast, injured-mino animals spent a comparable amount of time to animals in the two other groups did in the open and closed arms of the maze.

**Figure 3:**
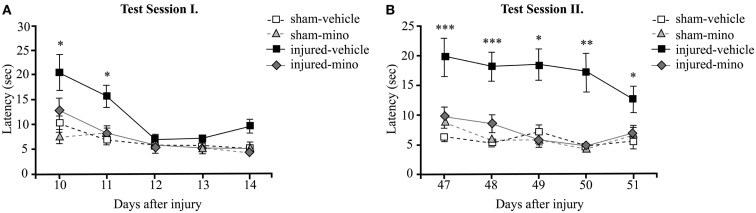
**The effect of injury and minocycline treatment on spatial memory at different time points after mbTBI**. Latency (seconds) to find and enter the escape box was measured for five consecutive days in the Barnes maze starting at **(A)** 10 days, and **(B)** 47 days after injury or sham. Data are presented as the average of the 2 daily trials per animal in each experimental group ±SEM. *p < 0.05, **p < 0.01, and ***p < 0.001 for injured-vehicle vs. sham-vehicle rats.

In order to assess time-dependent changes in spatial memory, we performed two tests in the BM at two different time points. Test Session I started at 10 days after injury and lasted for 5 days. Injured-vehicle animals performed poorly during the first 2 days of the test (Figure 3A). They required approximately twice as much time as animals in the other experimental groups to find the escape box. While their performance improved slightly on the second day of testing, injured-vehicle animals still required significantly more time to find the escape box compared to their sham group. On the third day of testing, their performance became roughly similar to animals in the other experimental groups. By contrast, the performance of injured-mino animals was very similar to uninjured (sham) animals; their measured latency times to locate and enter the escape box were almost identical on days 11 through 14. They found the escape box with slightly improved efficiency every day.

During Test Session II (beginning at 47 days post-injury), the performance of injured-vehicle animals was significantly worse than sham-vehicle animals on all five testing days (Figure 3B). While their performance slightly improved on each subsequent testing day, injured-vehicle rats still needed significantly more time to find the escape box, even on the last day of testing. Conversely, injured-mino animals performed similar to animals in the two control groups (sham-vehicle and sham-mino). Their performance during Test Session II was similar to that in Test Session I; they required about the same time to find the escape box on each testing day.

### Protein analyses

**Figure 4:**
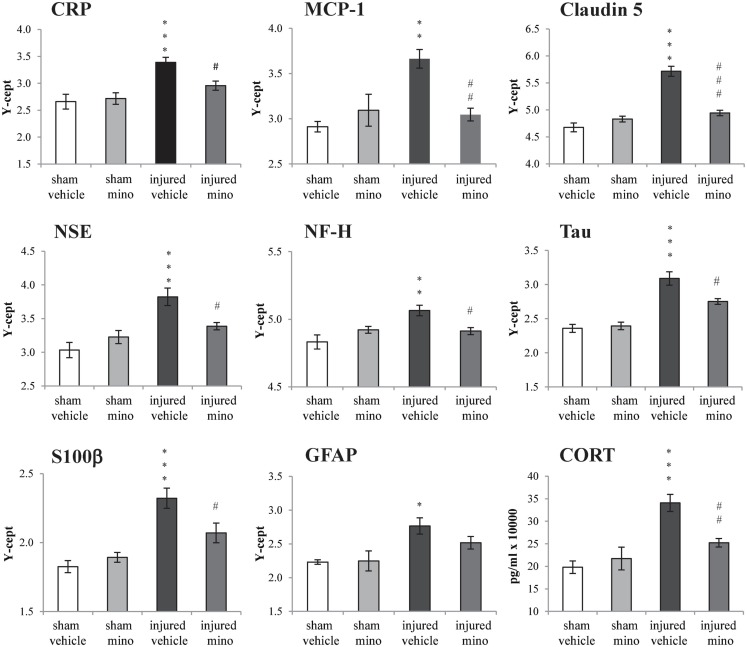
**The effect of injury and minocycline treatment on serum levels of selected markers in the different experimental groups**. Serum levels of 8 protein markers were assayed by RPPM; CORT levels were assayed by ELISA. Protein values are expressed as *y*-axis intercept (*Y*-cept) and CORT values are expressed as pg/ml. Data are presented as mean ± SEM. *p < 0.05, **p < 0.01, and ***p < 0.001 for injured-vehicle vs. sham-vehicle rats. ^#^*p* < 0.05, ^##^*p* < 0.01, and ^###^*p* < 0.001 for injured-vehicle vs. injured-mino rats.

Select protein marker levels were measured in the serum and dissected brain regions of animals in all four experimental groups. Injury without minocycline treatment caused a significant increase in the serum levels of all biomarkers measured (Figure 4). Both inflammatory markers, CRP and MCP-1, were significantly elevated in injured-vehicle animals; minocycline treatment resulted in normal or near normal (i.e., sham) sera levels in the injured-mino group. Claudin 5 levels were also elevated following blast injury in the vehicle-treated group, but were reduced to sham levels in injured-mino animals. Similarly, neuronal and glial loss and/or damage markers like NSE, NF-H, Tau, S100β, and GFAP were all significantly elevated in the sera of injured-vehicle animals. Minocycline treatment resulted in a significant reduction in serum levels of all of the markers except for GFAP. Lastly, serum CORT levels were also significantly increased in injured-vehicle rats, but minocycline treatment resulted in significantly lower serum CORT levels in injured-mino animals.

**Figure 5:**
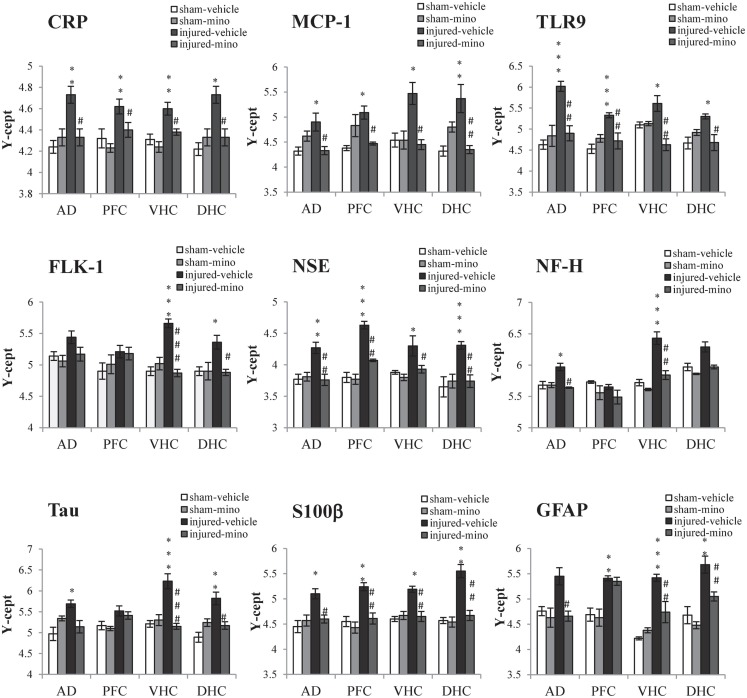
**The effect of injury and minocycline treatment on the levels of protein markers in various brain regions in the different experimental groups**. Tissue levels of 9 protein markers were measured in the AD, PFC, VHC, and DHC of rats by RPPM. Protein values are expressed as *y*-axis intercept (*Y*-cept) and data are presented as mean ± SEM. *p < 0.05, **p < 0.01, and ***p < 0.001 for injured-vehicle vs. sham-vehicle rats. ^#^*p* < 0.05 and ^##^*p* < 0.01 for injured-vehicle vs. injured-mino rats.

Tissue levels of 13 selected protein biomarkers (Figure 5; Table A3 in Appendix) were determined in the AD, PFC, VHC, and DHC of animals in the various experimental groups. We found significantly elevated levels of all three inflammatory markers (CRP, MCP-1, and TLR9) in the brains of injured-vehicle animals (Figure 5). Importantly, minocycline treatment of injured animals resulted in normal or near normal levels of these inflammatory markers; tissue levels of these markers in all four brain regions of injured-mino rats were not statistically different from those of sham-vehicle or sham-mino animals. NSE, S100β, and GFAP similarly showed injury-induced increases in all four brain regions. Minocycline treatment normalized their tissue levels with the exception of GFAP in the PFC, where GFAP levels of injured-vehicle and injured-mino animals were practically the same.

Some of the protein biomarkers that were analyzed showed brain region-dependent increases in response to injury. Of the vascular markers, tissue levels of FLK-1 (Figure 5), Claudin 5 and AQP4 (Table A3 in Appendix) were significantly elevated in the VHC following injury; FLK-1 and AQP4 levels were also elevated in the DHC and the AD, respectively. Similarly, neuronal and glial markers showed brain region-specific increases to injury. For instance, all three markers (NF-H, Tau, and MBP) showed injury-induced increases in the VHC but not in the PFC. Minocycline treatment of injured animals significantly reduced the tissue levels of all of the markers with the exception of Tau, which was not significantly reduced in the AD. Interestingly, VEGF did not show any significant changes in response to injury in any of the analyzed brain regions.

## Discussion

Minocycline is an FDA approved, semisynthetic, second-generation tetracycline drug that exhibits anti-inflammatory and/or neuroprotective effects in various experimental models of CNS disorders. These include focal and cerebral ischemia (Yrjanheikki et al., 1998; Xu et al., 2004), TBI (Sanchez Mejia et al., 2001), amyotrophic lateral sclerosis (Zhu et al., 2002), Parkinson’s disease (Wu et al., 2002), kainic acid treatment (Heo et al., 2006), Huntington’ disease (Chen et al., 2000; Du et al., 2001; Wu et al., 2002; Wang et al., 2003), multiple sclerosis (Brundula et al., 2002; Popovic et al., 2002), Alzheimer’s disease (Choi et al., 2007), and spinal cord injury (Wells et al., 2003; Stirling et al., 2004; Festoff et al., 2006; Table A1 in Appendix). Minocycline’s ability to improve outcome in distinct types of CNS disease models may stem from its ability to find multiple targets in different biochemical cascades that play a role in the development of the abovementioned diseases. Previous studies indicated that minocycline acts as a pleiotropic molecule; it can reduce the release of various chemokines and cytokines (Sanchez Mejia et al., 2001; Bye et al., 2007), lipid mediators of inflammation, matrix metalloproteinases (MMPs), and nitric oxide (NO; Stirling et al., 2005). Minocycline can also inhibit microglia activation (Yrjanheikki et al., 1998, 1999; Tikka and Koistinaho, 2001). The inhibition of microglial inflammatory responses has been reported in various neurodegenerative diseases (Yrjanheikki et al., 1999) including Huntington’s (Chen et al., 2000; Popovic et al., 2002; Wu et al., 2002); additional anti-inflammatory actions may be through the impediment of molecules like cyclooxigenase-2 (Patel et al., 1999; Yrjanheikki et al., 1999). Minocycline exerts its neuroprotective effects (Kriz et al., 2002; Wells et al., 2003; Stirling et al., 2004; Zemke and Majid, 2004; Marchand et al., 2009) through the repression of poly (ADP-ribose) polymerase-1 activity (Alano et al., 2006), which plays a central role in caspase-independent apoptosis (Susin et al., 1999; Zhang et al., 2002; Cao et al., 2003; Du et al., 2003), and the suppression of caspase-1 and caspase-3 expression (Chen et al., 2000) and cytochrome c release from the mitochondria (Zhu et al., 2002). Moreover, minocycline has been shown to sequester excess Ca^2+^ released after injury (Antonenko et al., 2010), and block the injury-induced decrease of soluble alpha amyloid precursor protein in the attenuation of diffuse axonal injury (Siopi et al., 2011). Based on all of these findings, we were compelled to test the effects of minocycline in our rat model of mbTBI.

During our pilot studies we followed a reported treatment schedule of 90 mg/kg of minocycline administered i.p. twice on the first day, 50 mg/kg twice per day for 2 subsequent days, and 50 mg/kg once per day for three additional days (Lee et al., 2003; Teng et al., 2004; Festoff et al., 2006; Yune et al., 2007). However, we found that this treatment caused substantial weight loss likely due to gastrointestinal problems (i.e., diarrhea). Based on these preliminary findings, we decided to modify the treatment paradigm by lowering the dose to 50 mg/kg once per day for four consecutive days. Our conservative treatment schedule caused light and transient diarrhea, and animals recovered and gained weight normally from the third day post-injury until the termination of the experiment on day 51 (data not shown).

Consistent with our previous findings, injured rats had reduced horizontal activity and a somewhat higher resting time than sham animals in the OF 1 day after injury (Kwon et al., 2011). Interestingly, all of the rats, independent of injury and treatment, showed gradually decreasing horizontal activities during the two subsequent OF sessions. There are two plausible explanations for this behavior. Rodents actively explore new areas, but inadvertently become less active on subsequent exposures to the same environment, a process called habituation (Pitkänen et al., 2006). We also observed on numerous occasions in other experiments that the horizontal activity of naïve rats in the OF at baseline is higher than it is 24 h later. We believe that since the OF represents a novel environment for the rats, they actively explore it (Bolivar et al., 2000; Daenen et al., 2001). However, repeated testing may cause the animals to habituate to the OF and in turn spend less time exploring and more time resting. Another possible explanation may be aging, especially during the last OF session, as young rodents have higher motor activity levels than more mature rodents (Sprott and Eleftheriou, 1974; Ingram et al., 1981; Gage et al., 1984; Lamberty and Gower, 1993). The effects of aging have also been observed as decreases in distance traveled in the EPM over time in both, sham and blast injured animals (Kovesdi et al., 2011).

Epidemiological studies have indicated that soldiers frequently develop neurobehavioral abnormalities like increased anxiety and memory impairments in mbTBI (Belanger et al., 2007; Brenner et al., 2009). Anxiety affects rehabilitation, psychosocial adjustment, and cognition in humans (Kersel et al., 2001; Rapoport et al., 2005). The EPM is a simple behavioral assay for evaluating the anxiety responses of rodents (Pellow et al., 1985) and studying the brain sites (limbic regions, hippocampus, amygdala; Silveira et al., 1993; Gonzalez and File, 1997) and the mechanisms underlying anxiolytic behavior (GABA, glutamate, serotonin, hypothalamic–pituitary–adrenal axis neuromodulators; Handley and Mithani, 1984; Pellow et al., 1985; Rodgers et al., 1992; Silva and Brandao, 2000; Korte and De Boer, 2003; Overstreet et al., 2003; Cortese and Phan, 2005). Rodents naturally prefer dark, enclosed spaces, and demonstrate an aversion to open spaces and a fear of heights (Barnett, 1975). Despite these natural inclinations, non-anxious rodents possess exploratory behaviors that cause them to investigate the open arms of the maze while more anxious rats remain in the closed arms of the maze for longer periods of time.

We previously found increased anxiety in our rodent model of mbTBI (Kovesdi et al., 2011). As our current EPM data illustrates, acute minocycline treatment prevented the increase in anxiety following blast overpressure. The time spent in the closed arms of the maze by injured-mino rats was indistinguishable from that of the two sham groups at both testing time points. Conversely, injured-vehicle animals showed signs of increased anxiety early on; they spent less time on the open arms of the maze than animals in the other three experimental groups. While the difference was not statistically significant at this early time point, injured-vehicle animals barely spent any time outside of the closed arms of the maze 46 days after the injury. Even though there is very little information available about the effects of minocycline on anxiety, especially in brain injury, minocycline treatment reduced anxiety in the EPM in models of cardiac arrest/cardiopulmonary resuscitation and fragile X syndrome (Bilousova et al., 2009; Neigh et al., 2009).

Current treatments of increased anxiety are mostly symptomatic (Tenovuo, 2006; Silver et al., 2009), and patients frequently experience side effects from the use of drugs like benzodiazepines (Rickels et al., 1991; Baldwin et al., 2005). Acute minocycline treatment may provide an alternative to the use of these drugs. Interestingly, injured-mino animals also had lower serum CORT levels than injured-vehicle animals at 51 days after the injury. While serum CORT levels have been used as indicators of stress (Dunn et al., 2004), the correlation between serum CORT levels and anxiety is rather complex and likely involve multiple regulatory pathways.

Consistent with available epidemiological data and our previous studies, the memory impairment associated with mbTBI develops over several weeks after the insult (Kovesdi et al., 2011; Kwon et al., 2011). Importantly, the deficit persists for at least 2 months post-injury (Kovesdi et al.). Given that 2 months in the lifespan of a rat roughly translates into several human years (Quinn, 2005), the observed memory impairment mirrors the chronic condition that manifests in humans reasonably well. The BM has been extensively used to study spatial learning and memory in rats (Barnes, 1979), and is considered a less anxiogenic alternative to the Morris water maze since it does not involve swimming (Pompl et al., 1999; Miyakawa et al., 2001; Deacon and Rawlins, 2002; Holmes et al., 2002). BM has been applied to studies of TBI; rodents with hippocampal damage show impaired performance in the maze, supporting the spatial nature of the task (Fox et al., 1998; Paylor et al., 2001; Deacon and Rawlins, 2002; Raber et al., 2004). In BM animals are presumed to learn the location of an escape hole using spatial reference points that are either fixed in relation to the maze (extra-maze cues) or are fixed on the maze itself in relation to the escape hole (proximal cues). It is important to note that during our acclimation and baseline behavioral testing, all animals were exposed to the maze and were trained to “learn” the task of locating and entering the escape box.

Early signs of the memory deficit were detected in the first testing session. Injured-vehicle animals required approximately twice as long to locate the escape box on the first day of testing, while injured-mino animals performed similar to the uninjured shams. On the second day of testing, injured-vehicle rats still needed more time than the other groups. During the last 3 days of testing, injured-vehicle rats relearned and remembered the task, requiring about the same amount of time as the other groups. However, during the second testing session, injured-vehicle rats performed poorly on all five testing days with only minor improvements in their speed from day to day. Conversely, injured-mino rats performed as well as sham animals did throughout. A similar effect was found in a study by Siopi et al. (2011) where acute treatment with minocycline significantly improved recognition memory; the effects lasted for up to 13 weeks in a mouse closed head injury model. There are currently no effective treatments in clinical use for memory impairment. Existing therapies predominantly target symptoms associated with mood disorders (e.g., depression) that can also improve memory performance (Tenovuo, 2006; Silver et al., 2009). Therefore, acute minocycline treatment has the potential to offer a potentially effective alternative.

The observed neurobehavioral impairments implicate the AD, PFC, VHC, and DHC due to their involvement in mediating anxiety and memory (Henke, 1990; Moser and Moser, 1998). In our earlier works we found indications of inflammation, axonal, glial, and neuronal damage in these brain regions (Kovesdi et al., 2011; Kwon et al., 2011). The neuroinflammatory response to various brain insults has been suggested as a potential link between injury and altered behavior, including increased anxiety. As reported earlier, blast can trigger a systemic inflammatory process even when the body is fully protected and only the head is exposed (Cernak et al., 2011). It is crucial to note that the similarities and the dissimilarities between mbTBI and other better-characterized forms of closed head injuries are currently not known with regards to their primary and secondary injury mechanisms. Nevertheless, it has been hypothesized that the different types of TBIs may share pathological components like neuroinflammation, neuronal and glial cell loss, and axonal injuries (Agoston et al., 2009).

In our current study, we found that minocycline treatment normalized significantly elevated sera levels of the inflammatory markers CRP and MCP-1 following exposure to mild blast. CRP and MCP-1 levels are routinely monitored in clinical settings and are used as an indicator of inflammation (Berman et al., 1996; Glabinski et al., 1996; Du Clos, 2000; Lobo et al., 2003). CRP is a component of the acute phase response to injury (Du Clos, 2000) and its expression is stimulated by the release of cytokines (Okamura et al., 1990); elevated CRP serum levels may reflect a combination of systemic as well as neuronal inflammation. Increased levels of MCP-1 are associated with neurological dysfunction after traumatic axonal injury in rats (Rancan et al., 2001), and are detected in the cerebrospinal fluid in diseases related to neuroinflammation such as stroke, meningitis, and multiple sclerosis (Mastroianni et al., 1998; Losy and Zaremba, 2001; Sindern et al., 2001; Chen et al., 2003; Sorensen et al., 2004). MCP-1 has also been suggested to regulate vascular permeability during CNS inflammation (Tekstra et al., 1999;Stamatovic et al., 2003, 2006).

While tissue levels of Claudin 5 did not significantly change except in the VHC, serum levels were significantly increased in injured-vehicle animals. Claudin 5 is a part of the tight junction complex in brain endothelial cells that contribute to the formation of the BBB (Morita et al., 1999; Liebner et al., 2000); increased serum levels suggest that there may be vascular damage in mbTBI that results in the release of Claudin 5 into systemic blood. Importantly, minocycline treatment normalized Claudin 5 sera levels indicating that vascular changes may be secondary to the inflammatory process or that minocycline possesses cytoprotective effects that also extend to endothelial cells.

Elevated serum levels of neuron- and glia-specific proteins have been found clinically as well as experimentally in various forms of TBI (Povlishock and Christman, 1995; Povlishock and Pettus, 1996; Buki and Povlishock, 2006). Increased serum levels of large neuron-specific molecules also point toward a vascular pathology; heightened BBB permeability is required for the release of large proteins like NF-H from the brain parenchyma and into systemic circulation. In a large animal model of blast TBI, the temporal pattern of serum NF-H levels correlated with clinical and pathological outcomes (Gyorgy et al., 2011). In our current study, minocycline treatment significantly reduced sera levels of NSE, NF-H, Tau, and S100β after injury, but not GFAP, an astroglia-specific intermediate filament (Missler et al., 1999) indicative of brain damage.

Consistent with our behavioral and serum data, we found that minocycline treatment prevented or mitigated injury-induced increases of the selected inflammatory markers CRP, MCP-1, and TLR9 in all four brain regions. TLR9 is member of the toll-like receptor family (Aderem and Ulevitch, 2000; Akira et al., 2001; Takeda and Akira, 2005; Mishra et al., 2006; O’Neill, 2006; Casanova et al., 2011) involved in the induction and the regulation of the inflammatory response in TBI (Hua et al., 2007, 2009) as well as other disorders involving neuroinflammation (Prat and Antel, 2005) and ischemic brain damage (Hua et al., 2007, 2009; Doyle et al., 2008; Gao et al., 2009; Marsh et al., 2009).

Of the vascular markers only FLK-1 and AQP4 tissue levels increased in response to the injury; minocycline treatment mitigated the effect of injury on FLK-1 levels but showed no effect on the tissue levels of AQP4. Increases in AQP4 were only detected in the AD and in the VHC while FLK-1 was in the VHC and the DHC. Elevations in AQP4 expression can contribute to the formation as well as the resolution of edema (Kimelberg, 1995; Papadopoulos et al., 2002; Amiry-Moghaddam and Ottersen, 2003; Neal et al., 2007). The pathology of severe bTBI includes the development of rapid and malignant brain edema (Ling et al., 2009; Ling and Ecklund, 2011) probably involving AQP4 (Neal et al., 2007). However, we currently have no information about water imbalance in mbTBI; if present, it is likely limited to the early phase following injury.

FLK-1 is a membrane-bound tyrosine kinase that mediates the effects of VEGF in the CNS (Sondell et al., 2000; Ogunshola et al., 2002; Rosenstein et al., 2003). Activation of FLK-1 stimulates various intracellular signal transduction pathways including the PI3K/Akt pathway that mediates the neuroprotective function of VEGF (Gerber et al., 1998; Wu et al., 2000; Kilic et al., 2006). VEGF/FLK-1 up-regulation following TBI seems to perform an important endogenous cytoprotective mechanism (Skold et al., 2006; Lee and Agoston, 2009). Interestingly, we did not detect changes in the abundance of VEGF in any of the analyzed brain regions following injury. A potential explanation for this negative finding is the relatively late testing time point (51 days post-injury). In a previous study using another model of TBI, we observed significant increases in VEGF tissue levels in the hippocampus (Lee and Agoston, 2009, 2010); the increases were limited to a few days after the injury.

The tissue levels of NSE, NF-H, Tau, S100β, GFAP, and MBP similarly increased in response to the injury, however, increases were brain region-specific. We measured significant injury-induced increases in sera levels of these proteins indicative of neuronal and glial cell losses. Thus, the detected increases in the tissue levels of these proteins are likely compensatory in nature and can be a part of the repair mechanism (Fawcett, 2009). Importantly, in all cases where injury resulted in an increase in the tissue levels of these markers, minocycline treatment mitigated the effect and tissue levels of these markers were restored to levels measured in sham animals.

## Conclusion

Our study demonstrates that acute minocycline treatment substantially improve the neurobehavioral outcome in a rodent model of mbTBI likely through mitigating the neuroinflammatory response to injury. The strength of our study lies in combining neurobehavioral tests performed at two different time points after injury with determining changes in serum and brain tissue levels of protein biomarkers. The limitations of the current study are the limited types of neurobehavioral and a single terminal time point of proteomics analyses. Based on these promising results, additional neurobehavioral testing shall be performed in future studies along with obtaining blood at several clinically relevant time points for protein assays. Nevertheless, our findings provide a rationale for exploring the viability of using acute minocycline treatment in mbTBI.

## Conflict of Interest Statement

The authors declare that the research was conducted in the absence of any commercial or financial relationships that could be construed as a potential conflict of interest.

## Appendix

**Table A1:** **List of animal models of various diseases, dose of minocycline treatment and the observed effects of the treatment**.

Animal model of disease	Dose	Effect	Reference
Acute spinal cord injury (mouse)	1 and 24 h (50 mg/kg, i.p.), then 25 mg/kg dose every 24 h for the next 5 days	Improved both hindlimb function and strength after injury and reduced lesion size	Wells et al. (2003)
Amyotrophic lateral sclerosis (mouse)	1 g/kg in a custom made rodent diet	Delayed the onset of motor neuron degeneration, less activation of microglia was detected at early symptomatic stage (46 weeks) and at the end stage of disease in the spinal cord	Kriz et al. (2002)
Cervical spinal cord injury (rat)	1 h (90 mg/kg), then for 3 days after injury	Failed to improve functional and histological recovery.	Lee et al. (2010)
Closed head injury (mouse)	5 min (90 mg/kg, i.p.), and at 3 and 9 h (45 mg/kg) post-TBI	Attenuation of the decrease of post-TBI sAPPα 24 h post-injury. Corpus callosum and striatal atrophy, ventriculomegaly, astrogliosis, and microglial activation reduced 3 months post-injury	Siopi et al. (2011)
Closed head injury (mouse)	30 min (45 mg/kg, i.p.) and every 12 h (22.5 mg/kg, i.p.) for 1 week. Or twice-daily minocycline injections for 2 weeks (6 weeks surviving)	Reduced the activation of microglia/macrophages and improved neurological outcome, but any increase of neurogenesis	Ng et al. (2012)
Controlled contusion spinal cord injury (rat)	Multiple injections (30 mg/kg, i.p.) at 0.5, 1, and 24 h, or a single injection of 90 mg/kg at either 0.5, 1.0, or 24 h after injury	Improved functional recovery, reduced tissue damage, cavity size, apoptosis and activated caspase-3 signal	Festoff et al. (2006)
Controlled cortical impact (rat)	45 mg/kg, i.p. at 1 h, 24 and 48 h after injury	Improved active place avoidance following CCI	Abdel Baki et al. (2010)
Endothelin-1 (ET-1) model of focal ischemia (rat)	45 mg/kg, i.p. at 2 and 12 h following the last injection of ET-1, then 22.5 mg/kg every 12 h (5×)	Improved behavioral outcome. Reduced subcortical and whole hemisphere infarct volume	Hewlett and Corbett (2006)
Focal cerebral ischemia (rat)	45 mg/kg, i.p. twice a day for the first day; 22.5 mg/kg for the subsequent 2 days	Reduced cortical infarction volume, inhibited morphological activation of microglia in the area adjacent to the infarction, induction of IL-1b-converting enzyme, and reduced cyclooxygenase-2 expression and prostaglandin E2 production	Yrjanheikki et al. (1999)
Huntington disease (mouse)	daily 5 mg/kg, i.p.	Inhibited caspase-1 and caspase-3 up-regulation	Chen et al. (2000)
Middle cerebral artery occlusion (MCAO; mice)	45 mg/kg two times in every 12 h starting at 30 min after the onset of MCAO	Neuroprotectant at males, but ineffective at reducing ischemic damage in females	Li and McCullough (2009)
Neonatal hypoxia-ischemia (HI; rat)	2 h after hypoxia (45 mg/kg, i.p.), then every 24 h from P4–P9 (22.5 mg/kg)	Prevention of HI induced changes in SERT, 5-HT and 5-HT positive dorsal raphe neurons. Lasting effect after 6 week of HI	Wixey et al. (2011)
Parkinson disease (mouse)	Daily twice (12 h apart) injections from 1.4 to 45 mg/kg (i.p.) starting 30 min after the first MPTP injection and continuing through four additional days after the last injection of MPTP	Inhibited microglial activation, mitigated both the demise of nigrostriatal dopaminergic neurons and the formation of nitrotyrosine. Prevented the formation of mature interleukin-1β and the activation of NADPH–oxidase and inducible nitric oxide synthase (iNOS)	Wu et al. (2002)
Spinal cord injury (T13 hemisection of the spinal cord; rat)	30 min (40 mg/kg, i.p.) followed twice per day for 2 days post-injury	Reduced the development of pain behaviors at 1 and 2 weeks after SCI, reduced microglial OX-42 expression and decreased the expression of noxious stimulation-induced c-Fos	Marchand et al. (2009)
Spinal cord injury (rat)	Twice a day beginning 30 min after injury (50 mg/kg, i.p.) for 2 days	Reduced apoptotic oligodendrocytes and microglia in proximal and distal segments of the ascending sensory tract. Reduced microglial/macrophage density, attenuated axonal dieback and improved functional outcome	Stirling et al. (2004)
Temporary middle cerebral artery occlusion model (TMCAO; rat)	For 4 h post TMCAO protocol: 3 or 10 mg/kg i.v. at 4, 8, and 12 h; for the 5-h post TMCAO protocol: at 5, 9, and 13 h; and for the 6-h post TMCAO protocol at 6, 10, and 14 h	3 and 10 mg/kg i.v. were effective at reducing infarct size with a 5 hour therapeutic time window after TMCAO. 10 mg/kg extended the window time to ameliorate neurological deficits to 5 h	Xu et al. (2004)

**Table A2:** **List of antibodies and their respective classifications and dilutions used to measure protein biomarker levels in sera and brain tissues**.

Antibody	Vendor	Catalog No.	Dilution in RPPM
**INFLAMMATORY**			
C-reactive protein (CRP)	Santa Cruz Biotechnology, Inc.	sc-30047	1:20
Monocyte chemoattractant protein (MCP-1)	Santa Cruz Biotechnology, Inc.	sc-1784	1:20
*Toll-like receptor 9 (TLR9)*	*Santa Cruz Biotechnology, Inc*.	*sc-13218*	*1:20*
**VASCULAR**			
Claudin 5	Santa Cruz Biotechnology, Inc.	sc-28670	1:20
*Vascular endothelial growth factor (VEGF)*	*Abcam*	*ab-53465*	*1:50*
*VEGF receptor 2 (FLK-1)*	*Santa Cruz Biotechnology, Inc*.	*sc-315*	*1:20*
*Aquaporin 4 (AQP4)*	*Abcam*	*ab-97414*	*1:50*
**NEURONAL**			
Neuron-specific enolase (NSE)	Abcam	ab-53025	1:20
Neurofilament heavy chain (NF-H)	Sigma Aldrich	N-4142	1:20
Tau protein	Santa Cruz Biotechnology, Inc.	sc-1995P	1:20
**GLIAL**			
S100 beta protein (S100β)	Abcam	ab-41548	1:20
Glial fibrillary acidic protein (GFAP)	Abcam	ab-7260	1:50
*Myelin basic protein (MBP)*	*Santa Cruz Biotechnology, Inc*.	*sc-13914*	*1:20*

*Biomarkers labeled with *italics* were only measured in the brain*.

**Table A3:** **The effect of injury and minocycline treatment on tissue levels of the selected protein biomarkers in the different experimental groups**.

Markers	Amygdala				Prefrontal cortex				Ventral hippocampus				Dorsal hippocampus			
	Sham vehicle	Sham-mino	Injured-vehicle	Injured-mino	Sham vehicle	Sham-mino	Injured-vehicle	Injured-mino	Sham vehicle	Sham-mino	Injured-vehicle	Injured-mino	Sham vehicle	Sham-mino	Injured-vehicle	Injured-mino
**VASCULAR**																
**Claudin 5**	4.49 ±	4.61 ±	4.90 ±	4.81 ±	4.25 ±	4.40 ±	4.63 ±	4.75 ±	4.10 ±	4.38 ±	*4.78 *±	4.59 ±	4.65 ±	4.61 ±	4.64 ±	4.59 ±0.06
	0.14	0.09	0.18	0.14	0.16	0.15	0.05	0.08	0.09	0.14	*0.15*	0.08	0.12	0.12	0.18	0.06
**VEGF**	4.97 ±	5.15 ±	5.30 ±	4.97 ±	5.07 ±	4.83 ±	5.12 ±	4.97 ±	5.03 ±	4.90 ±	5.18 ±	5.10 ±	4.99 ±	5.01 ±	4.89 ±	5.00 ±0.15
	0.06	0.07	0.11	0.10	0.05	0.06	0.07	0.12	0.04	0.06	0.07	0.04	0.08	0.08	0.16
**AQP4**	4.36 ±	4.44 ±	*4.78 *±	4.53 ±	4.36 ±	4.37 ±	4.58 ±	4.68 ±	4.25 ±	4.30 ±	*4.80 *±	4.49 ±	4.32 ±	4.36 ±	4.41 ±	4.41 ±0.12
	0.05	0.08	*0.04*	0.07	0.12	0.11	0.05	0.13	0.07	0.10	*0.04*	0.07	0.09	0.07	0.08
**GLIAL**																
**MBP**	5.63 ±	5.55 ±	5.64 ±	5.49 ±	5.54 ±	5.64 ±	5.93 ±	5.94 ±	6.00 ±	*6.37 *±	**6.05 ±**	5.94 ±	6.04 ±	*6.51 *±	**5.95** ±
	0.14	0.06	0.08	0.09	0.06	0.06	0.04	0.15	0.08	0.04	*0.11*	**0.04**	0.07	0.08	*0.04*	**0.11**

*The levels of seven protein markers were measured by RPPM in the amygdala, prefrontal cortex, ventral hippocampus, and dorsal hippocampus. Measured protein levels are expressed as *y*-axis intercept (*Y*-cept) and are presented as mean ± SEM. Values listed in *underline italics* indicate *p* < 0.05 for injured-vehicle vs. sham-vehicle rats. **Bold-faced** values indicate *p* < 0.05 for injured-vehicle vs. injured-minorats*.
